# DMH1 Increases Glucose Metabolism through Activating Akt in L6 Rat Skeletal Muscle Cells

**DOI:** 10.1371/journal.pone.0107776

**Published:** 2014-09-23

**Authors:** Xin Xie, Xiao-Ming Xu, Na Li, Yong-Hui Zhang, Yu Zhao, Chun-Yan Ma, De-Li Dong

**Affiliations:** Department of Pharmacology (the State-Province Key Laboratories of Biomedicine-Pharmaceutics of China, Key Laboratory of Cardiovascular Research, Ministry of Education), Harbin Medical University, Harbin, P.R. China; The Chinese University of Hong Kong, Hong Kong

## Abstract

DMH1(4-[6-(4-Isopropoxyphenyl)pyrazolo [1,5-a]pyrimidin-3-yl] quinoline) is a compound C analogue with the structural modifications at the 3- and 6-positions in pyrazolo[1,5-a]pyrimidine backbone. Compound C was reported to inhibit both AMPK and Akt. Our preliminary work found that DMH1 activated Akt. Since Akt was involved in glucose metabolism, we aimed to identify the effects of DMH1 on glucose metabolism in L6 rat muscle cells and the potential mechanism. Results showed that DMH1 increased lactic acid release and glucose consumption in L6 rat muscle cells in a dose-dependent manner. DMH1 activated Akt in L6 cells. Akt inhibitor inhibited DMH1-induced Akt activation and DMH1-induced increases of glucose uptake and consumption. DMH1 had no cytotoxicity in L6 cells, but inhibited mitochondrial function and reduced ATP production. DMH1 showed no effect on AMPK, but in the presence of Akt inhibitor, DMH1 significantly activated AMPK. Compound C inhibited DMH1-induced Akt activation in L6 cells. Compound C inhibited DMH1-induced increase of glucose uptake, consumption and lactic acid release in L6 cells. DMH1 inhibited PP2A activity, and PP2A activator forskolin reversed DMH1-induced Akt activation. We concluded that DMH1 increased glucose metabolism through activating Akt and DMH1 activated Akt through inhibiting PP2A activity in L6 rat muscle cells. In view of the analogue structure of DMH1 and compound C and the contrasting effects of DMH1 and compound C on Akt, the present study provides a novel leading chemical structure targeting Akt with potential use for regulating glucose metabolism.

## Introduction

Compound C is an AMP-activated protein kinase (AMPK) inhibitor and is widely used in the studies related to AMPK. For example, it was reported that AMPK inhibition by compound C prevented glucose uptake induced by testosterone in cardiomyocytes [1], attenuated LPS-induced immune responses and liver injury [2]. However, several studies have shown that compound C also exerted its action in an AMPK-independent manner. Vucicevic *et al*. found that compound C induced autophagy in cancer cells through AMPK inhibition-independent blockade of Akt/mTOR pathway [3]. DMH1(4-[6-(4-Isopropoxyphenyl)pyrazolo [1,5-a]pyrimidin-3-yl] quinoline) was a compound C analogue with the structural modifications at the 3- and 6-positions in pyrazolo[1,5-a]pyrimidine backbone [4]. DMH1 was initially developed as a bone morphogenetic protein (BMP) selective inhibitor [4] and our preliminary work showed that DMH1 activated Akt in L6 cells.

Protein kinase B (Akt) and AMPK play important roles in regulating the glycolysis process. Akt is a serine/threonine-specific protein kinase involved in multiple cellular processes such as glucose metabolism and cell proliferation. AMPK regulates metabolic pathways to balance energy at both cellular and whole-body levels [5], [6]. Since Akt was involved in glycolysis, we aimed to identify the effects of DMH1 on glucose metabolism in L6 rat muscle cells that are generally used in glycolysis studies. Because both DMH1 and compound C have the similar chemical structure, we further compared the effects of compound C with DMH1, looking forward to providing a novel clue on development of drugs regulating glucose metabolism based on the structure and pharmacological effects of DMH1 and compound C.

## Materials and Methods

### Agents

Dulbecco's modified Eagle's medium (DMEM), fetal bovine serum (FBS) were purchased from Hyclone. DMH1, compound C, Akt1/2 kinase inhibitor (1,3-Dihydro-1-(1-((4-(6-phenyl-1H-imidazo[4,5-g]quinoxalin-7-yl)phenyl)methyl)-4-piperidinyl)-2H-benzimidazol-2-one trifluoroacetate salt hydrate)and insulin were obtained from Sigma-Aldrich. Rat L6 skeletal muscle cells were purchased from the Type Culture Collection of the Chinese Academy of Sciences, Shanghai, China. Live/dead Viability/Cytotoxicity Assay kit was obtained from Invitrogen. DMEM glucose free was obtained from Gibco. Anti-phospho-Akt Ser473, anti-Akt, anti-phospho-AMPK Thr172, anti-AMPK and PP2A antibodies were obtained from Cell Signaling Technology. Glucose uptake cell-based assay kit was obtained from Cayman Chemical Company. Glucose assay kit, lactic acid assay kit, and lactate dehydrogenase (LDH) assay kit were purchased from Nanjing Jiancheng Bioengeineering Institute (China). ATP assay kit and PP2A agonist forskolin were purchased from Beyotime Institute of Biotechnology (China).

### Cell culture

L6 skeletal muscle cells were grown in Dulbecco's modified Eagle's medium containing 5.5 mmol/l glucose and supplemented with 10% fetal bovine serum, 100 units/ml penicillin and 100 µg/ml streptomycin at 37°C, 5%CO_2_. The time of treatment and the concentration of agents were shown in figures and/or figure legends.

### Measurement of glucose consumption and lactic acid content

The cells were grown in 6-well plates. After the indicated periods of incubation with different treatments, the culture medium was collected; the glucose concentration in the medium was determined by the glucose oxidase method (Nanjing Jiancheng Biological Engineering Research Institute, China). The amount of lactic acid in the medium was measured by a commercial assay according to the instruction (Nanjing Jiancheng Biological Engineering Research Institute, China). In brief, pyruvic acid was produced from lactic acid with reduction of NAD^+^ to NADH. Simultaneously, the tetranitroblue tetrazolium chloride (NBT) was also reduced to purple colored substances whose absorbance was read at 530 nm using a plate reader.

### Glucose uptake assay

Cells (5×10^4^ cells) were seeded in a 96-well black and clear bottom plate with 100 µl culture medium. After incubation at 37°C, 5%CO_2_ overnight, the medium was replaced with glucose-free medium containing 150 µg/ml 2-NBDG (a fluorescently-labeled deoxyglucose analog). Then cells were exposed to DMH1 or compound C, or Akt1/2 kinase inhibitor for 24 hrs. The plate was centrifuged for 5 min at 400×g at room temperature, the supernatant was aspirated, and then 200 µl cell-based assay buffer was added and centrifuged again. Next, the supernatant was aspirated and 100 µl cell-based assay buffer was added again. The fluorescein at a wavelength of excitation/emission  = 485/535 nm was measured with a plate reader (Tecan Infinite m200, Mannedorf, Switzerland).

### Measurement of intracellular ATP and SDH activity

Cells were washed twice with PBS solution and then resolved in lysis buffer on ice. ATP was measured by luminometric methods using commercially available luciferin/luciferase reagents according to the manufacturer's instructions (ATP Assay Kit; Beyotime Biotech). Finally, ATP concentration was normalized to the protein levels. Succinatedehydrogenase (SDH) activity was measured by MTT assay. Cells were treated with different concentrations of DMH1 for 24 hrs and then incubated with 5 mg/ml MTT for 4 hrs at 37°C. Medium was then removed and 200 µl of DMSO was added to dissolve the crystal. Absorbance was measured at a wavelength of 490 nm with a plate reader (Tecan Infinite m200, Mannedorf, Switzerland).

### Lactate dehydrogenase (LDH) activity assay

Cells were treated with different concentration of DMH1 for 24 hrs, then cell culture medium was collected for LDH determination. LDH could catalyze the synthesis of pyruvic acid from lactic acid and then pyruvic acid reacted to form 2,4-dinitrophenyl-hydrazine which showed brownish red color in basic solution. After the reaction, the absorbance was read at wavelength 450 nm.

### Live and dead cell staining

The LIVE/DEAD Viability/Cytotoxicity Assay Kit (Invitrogen) was used to detect live and dead cells as described in our previous work [7]. Briefly, cells were grown on coverslips at a density of 3.75×10^4^/ml and incubated overnight at 37°C in a humidified 5%CO_2_ incubator. The cells were washed with PBS and dyed according to the manufacturer's instructions. The labeled cells were photographed under a fluorescence microscope. The live cells fluoresce green and dead cells fluoresce red.

### Western blot analysis

Detailed information was described in our previous works [7], [8]. Western blot bands were quantified by using Odyssey infrared imaging system (LI-COR) and Odyssey v3.0 software.

### PP2A activity assay

PP2A activity was determined by using the PP2A Immunoprecipitation Phosphatase Assay Kit (Millipore, US), assessed by dephosphorylation of the phosphopeptide (K-R-pT-I-R-R).

### Data analysis

Data are presented as mean±SEM. Significance was determined by using Student t test. *P*<0.05 was considered significant.

## Results

### DMH1 increases lactic acid release

**Figure 1:**
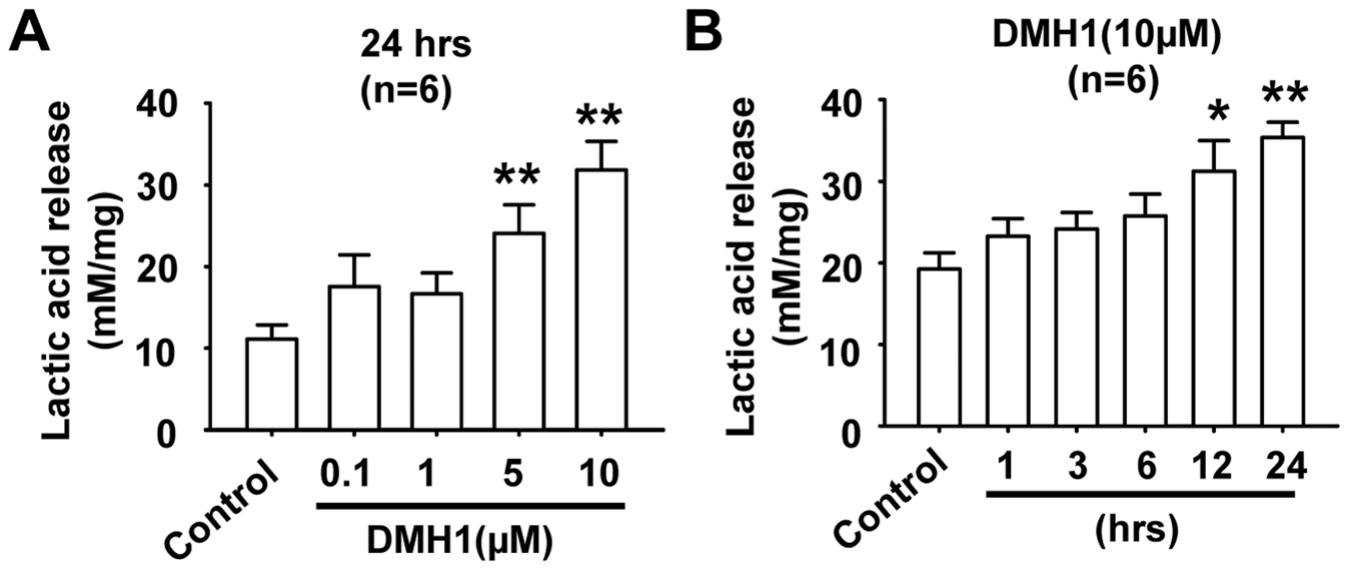
Dose- and time- dependent stimulation of lactic acid release in L6 cells by DMH1. (**A**) L6 cells were incubated with incremental concentrations of DMH1 for 24 hrs. n = 6. (**B**) L6 cells were incubated with 10 µM DMH1 at different incubation periods up to 24 hrs. n = 6. *P<0.05, **P<0.01 vs control.

Firstly, we measured the effects of DMH1 on lactic acid release in L6 rat muscle cells. As shown in Figure.1A, the lactic acid concentration was increased in the culture medium in a dose-dependent manner by DMH1 treatment for 24 hrs. Time course results indicated that 10 µM DMH1 increased lactic acid release at 12 hrs after DMH1 treatment (Figure.1B). Lactic acid is the product of anaerobic glycolysis. These results indicated that DMH1 stimulated anaerobic glycolysis.

### DMH1 increases glucose consumption

**Figure 2:**
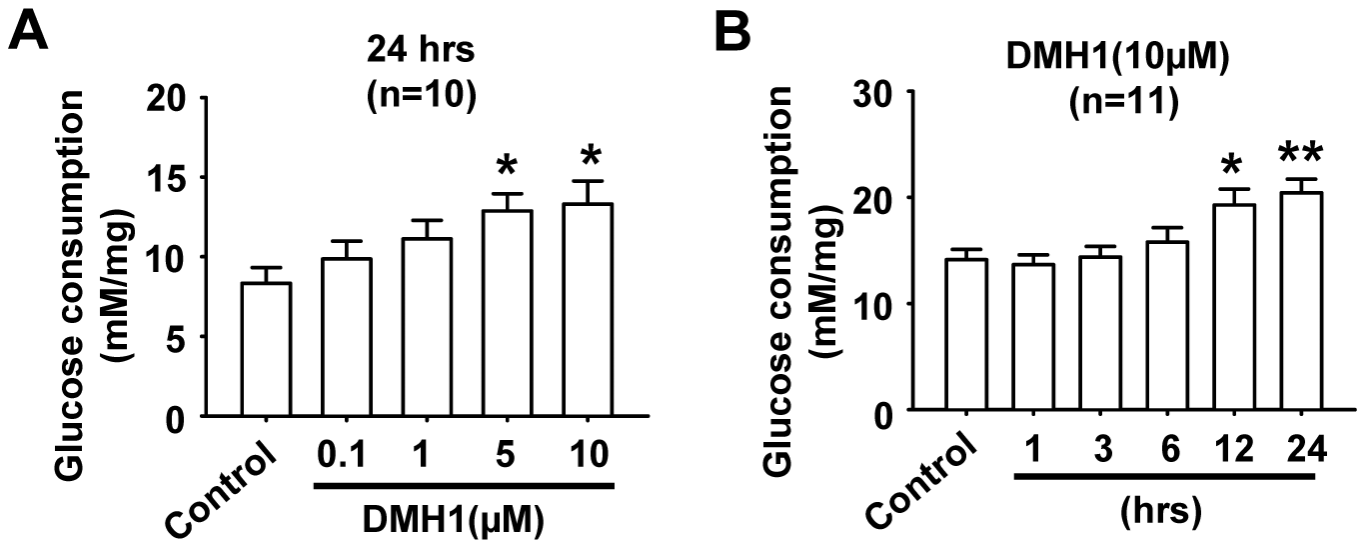
DMH1 increases glucose consumption in a dose- and time-dependent manner in L6 cells. (**A**) L6 cells were incubated with 0.1 µM,1 µM,5 µM and 10 µM DMH1 for 24 hrs. n = 10. (**B**) L6 cells were incubated with 10 µM DMH1 for 1, 3, 6, 12 and 24 hrs. Glucose consumption was normalized to total protein level. n = 11.*P<0.05, **P<0.01 vs control.

Next, we measured the effects of DMH1 on glucose consumption in L6 rat muscle cells. As shown in Figure.2A, DMH1 treatment increased glucose consumption in a dose-dependent manner. Time course results indicated that 10 µM DMH1 increased glucose consumption at 12 hrs after DMH1 treatment (Figure.2B). Based on the above data, we used DMH1 at 10 µM concentration and the time of DMH1 treatment was set as 24 hrs.

### DMH1 activates Akt in L6 cells

**Figure 3:**
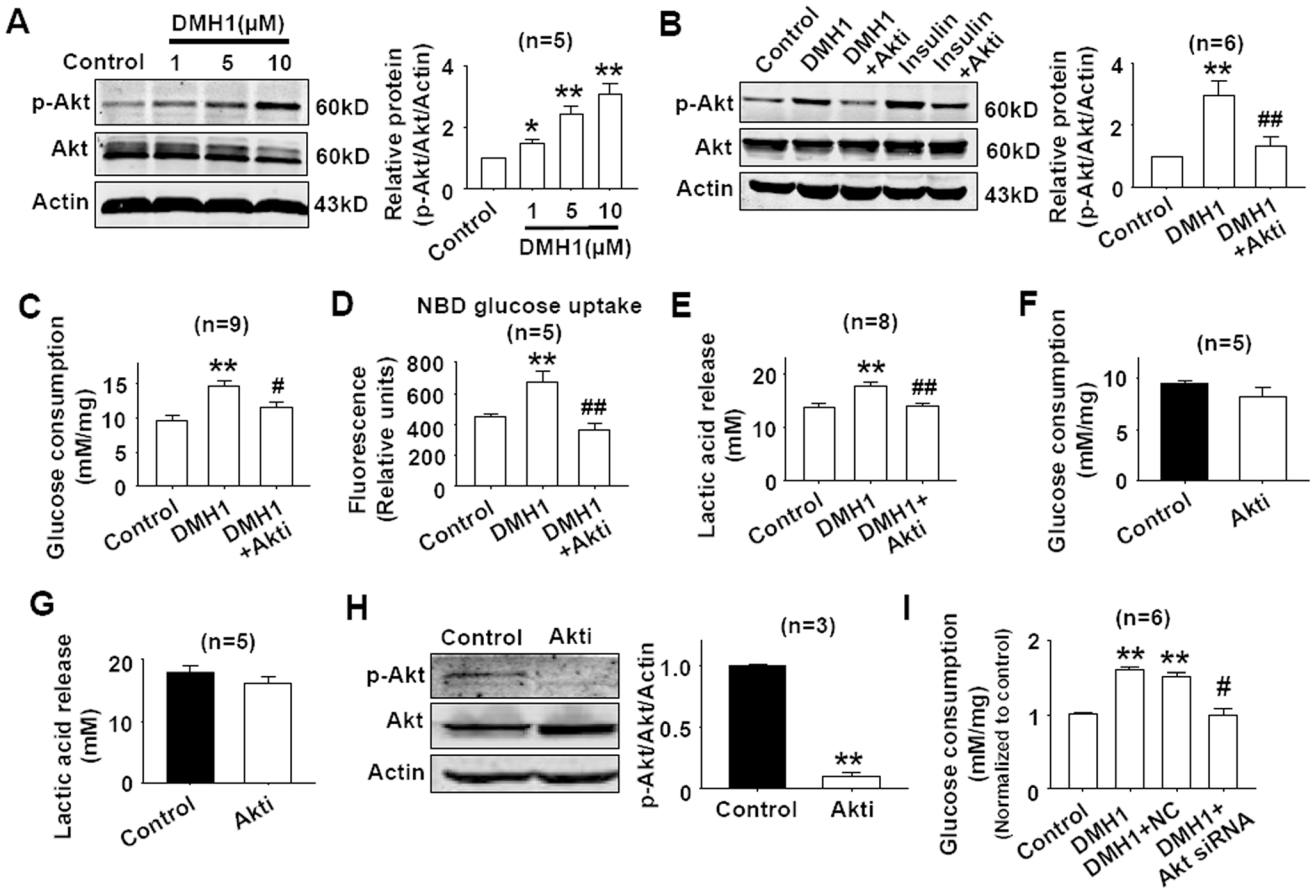
Effects of DMH1 on the phosphorylation of Akt. (**A**) DMH1 activated Akt in L6 cells. L6 cells were treated with 1 µM, 5 µM and 10 µM DMH1 for 24 hrs. n = 5. *P<0.05, **P<0.01 vs controls. (**B**) Akt inhibitor inhibited DMH1-induced Akt activation. L6 cells were preincubated with 0.5 µM Akt inhibitor for 1 hr before incubated with 10 µM DMH1 for 24 hrs. Whole cell lysate was detected for p-Akt through immunoblotting. n = 6. **P<0.01 vs control. ##*P*<0.01 vs DMH1. The concentration of insulin was 100 nM, and the incubation period was 30 min. (**C–E**) Akt inhibitor (0.5 µM) inhibited DMH1(10 µM)-induced glucose consumption, glucose uptake, and lactic acid release in L6 cells. **P<0.01 vs control. #*P*<0.05, ##*P*<0.01 vs DMH1. (**F–H**) Akt inhibitor (0.5 µM) alone showed no significant effects on glucose consumption and lactic acid release, but inhibited p-Akt level in L6 cells. Akti, Akt1/2 kinase inhibitor, (1,3-Dihydro-1-(1-((4-(6-phenyl-1H -imidazo[4,5-g]quinoxalin-7-yl)phenyl)methyl)-4-piperidinyl)-2H-benzimidazol-2-one trifluoroacetate salt hydrate). **P<0.01 vs control. (**I**) Akt siRNA inhibited DMH1-induced increase of glucose consumption in L6 cells. **P<0.01 vs control. #*P*<0.05, vs DMH1+NC. NC, negative control.

Compound C, a DMH1 analogue, inhibited Akt [3]. Here, we examined the effects of DMH1 on Akt in L6 cells. As shown in Figure.3A, DMH1 stimulated the phosphorylation of Akt in a dose-dependent manner. Akt inhibitor inhibited DMH1-induced Akt activation (Figure.3B). Meanwhile, the positive control insulin significantly stimulated the phosphorylation of Akt which was also inhibited by Akt inhibitor (Figure.3B). Since Akt activation was involved in glucose uptake and consumption [9], [10], we measured the effects of Akt inhibitor on the increased glucose uptake, consumption and lactic acid release induced by DMH1 treatment. As shown in Figure.3C–3E, DMH1-induced increase of glucose uptake, consumption and lactic acid release was inhibited by Akt inhibitor, indicating that DMH1 increased glucose uptake, consumption and lactic acid release through activating Akt in L6 cells. Akt inhibitor (0.5 µM) alone showed no significant effects on glucose consumption and lactic acid release, though Akt inhibitor inhibited p-Akt level in L6 cells (Figure.3F–3H). We further used Akt siRNA which had been proved to knockdown Akt expression in our previous work [11], to test the effect of DMH1 on glucose consumption. Results showed that Akt siRNA inhibited DMH1-induced increase of glucose consumption in L6 cells (Figure.3I).

### DMH1 has no cytotoxicity in L6 cells

**Figure 4:**
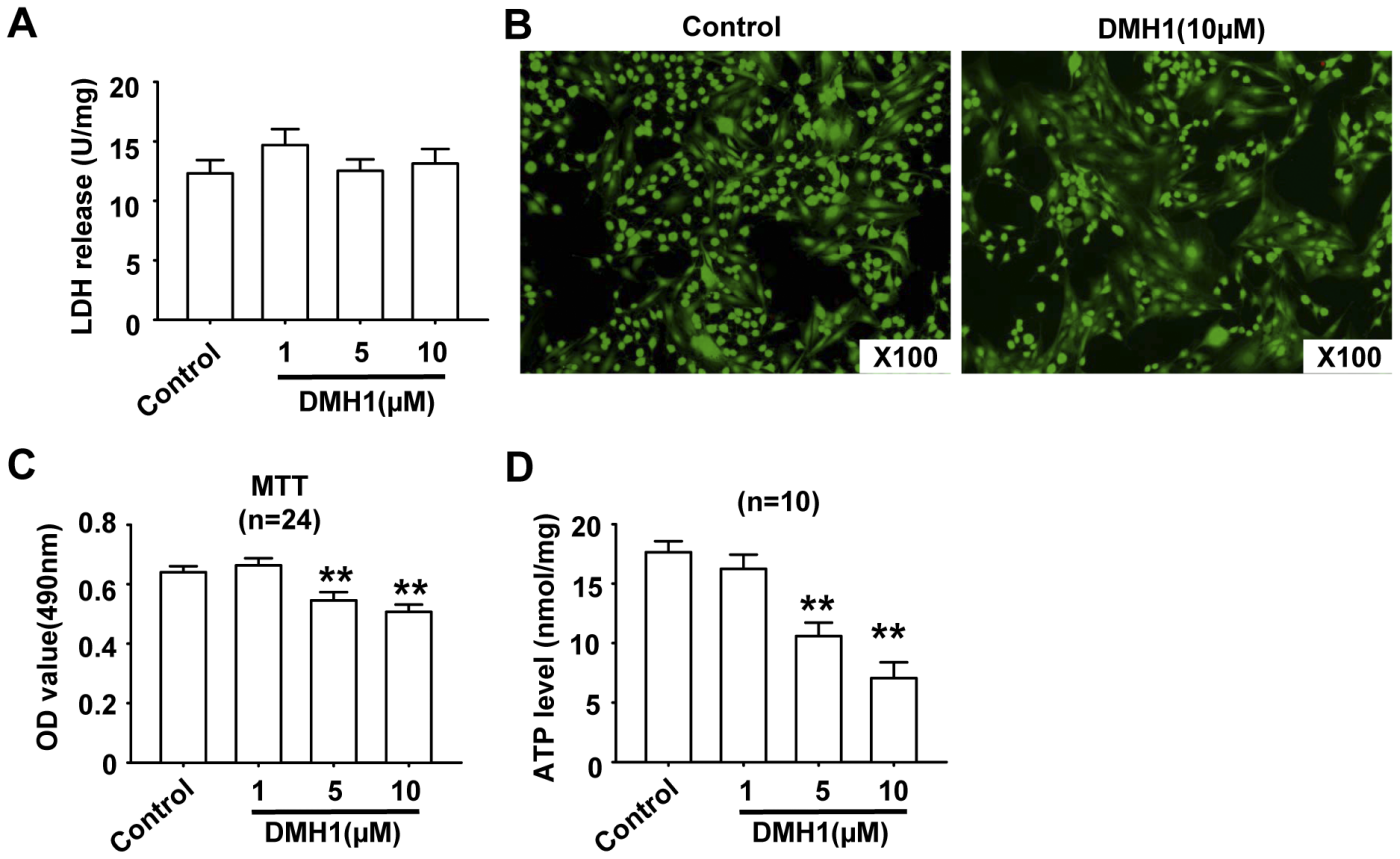
DMH1 has no cytotoxicity, but inhibited SDH activity and ATP production. (**A**) DMH1 did not increase LDH release in L6 cells. Cells were incubated with 1 µM, 5 µM and 10 µM DMH1 for 24 hrs. Then LDH units were normalized to total protein content. n = 5. (**B**) Representative photographs showing that DMH1 did not induce cell death. Cells were treated with 10 µM DMH1 for 24 hrs. The labeled cells were photographed under a fluorescence microscope. The live cells fluoresce green and dead cells fluoresce red. (**C**) MTT results showed that DMH1 inhibited succinate dehydrogenase (SDH) activity. n = 24. **P<0.01 *vs* control. (**D**) DMH1 treatment reduced ATP production in L6 cells. n = 6. **P<0.01 *vs* control.

In this study, the cells were treated with DMH1 for 24 hrs, so it was necessary to examine whether DMH1 had cytotoxicity in L6 cells. LDH cytotoxicity assay and LIVE/DEAD viability assay were used to address this issue. As shown in Figure.4A, DMH1 treatment did not increase LDH release during exposure to 1, 5, 10 µM DMH1 for 24 hrs. LIVE/DEAD viability assay results also showed that DMH1 had no cytotoxicity in L6 cells (Figure.4B). MTT assay was commonly used to evaluate mitochondrial succinate dehydrogenase activity, based on the fact that viable cells can reduce 3-(4,5-dimethylthiazol-2-yl)-2,5-diphenyl tetrazolium bromide (MTT). Succinate dehydrogenase is a marker enzyme reflecting the mitochondrial function for producing ATP [12], [13]. Since DMH1 had no cytotoxicity in L6 cells, MTT results indicated that DMH1 inhibited mitochondrial function (Figure.4C). Indeed, DMH1 reduced ATP levels in L6 cells in a dose-dependent manner (Figure.4D).

**Figure 5:**
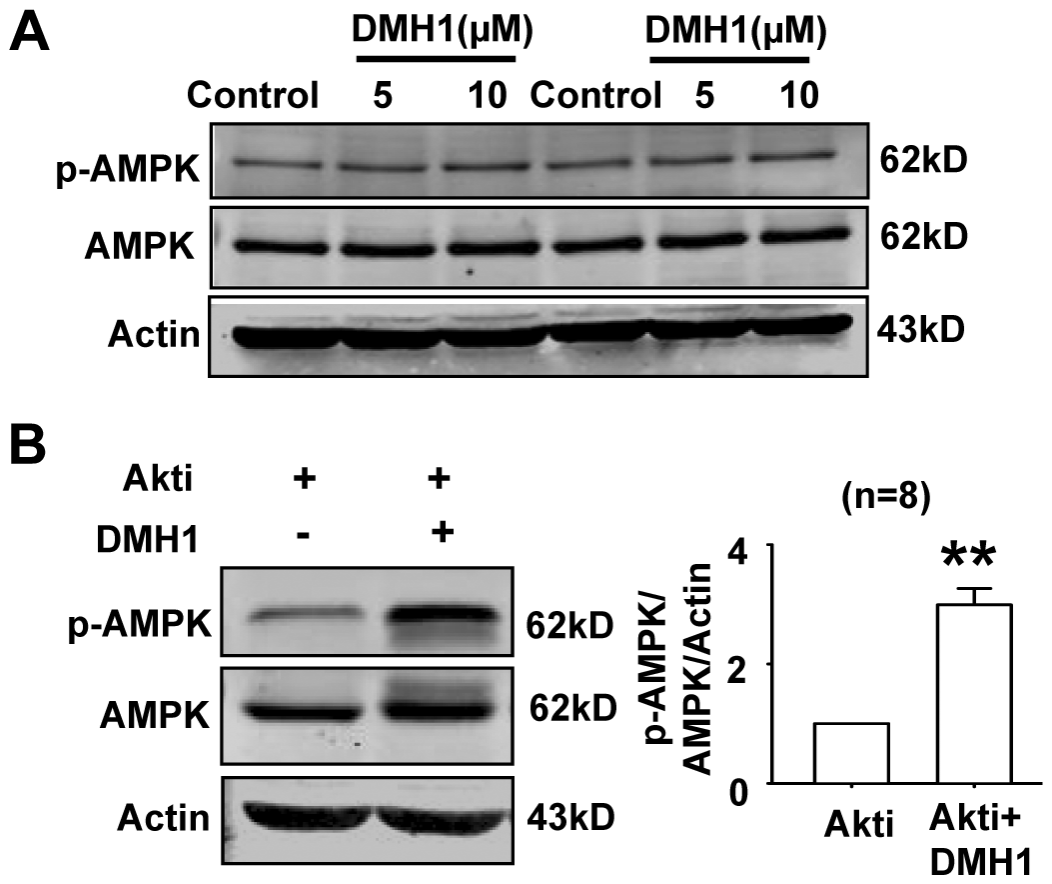
Effects of DMH1 on the phosphorylation of AMPK. (**A**) Representative western blot results showed that DMH1 did not affect phosphorylation of AMPK in L6 cells. The western blot represented three individual experiments. (**B**) DMH1(10 µM) activated AMPK in the presence of Akt inhibitor (0.5 µM). **P<0.01 *vs* Akti. Akti, Akt inhibitor, (1,3-Dihydro-1-(1-((4-(6-phenyl-1H -imidazo[4,5-g]quinoxalin-7-yl)phenyl)methyl)-4-piperidinyl)-2H-benzimidazol-2-one trifluoroacetate salt hydrate).

Activation of AMPK is often a consequence of a decrease of ATP production or an increase of AMP/ATP ratio [5], [14]. Since DMH1 reduced ATP levels in L6 cells, it would be expected that DMH1 could activate AMPK. However, we did not detect the significant activation of AMPK in L6 cells treated with DMH1 (Figure.5A). Next, we treated L6 cells with DMH1 in the presence of Akt inhibitor. Results showed that DMH1 significantly activated AMPK in the presence of Akt inhibitor (Figure.5B), indicating that DMH1 activated AMPK when Akt was inhibited by Akt inhibitor. Akt is a negative regulator of AMPK [15], [16], we speculated that AMPK activated by DMH1-induced decrease of ATP could be inhibited by DMH1-induced activation of Akt, so the activation of AMPK was not observed.

### Compound C inhibits DMH1-induced Akt activation in L6 cells

**Figure 6:**
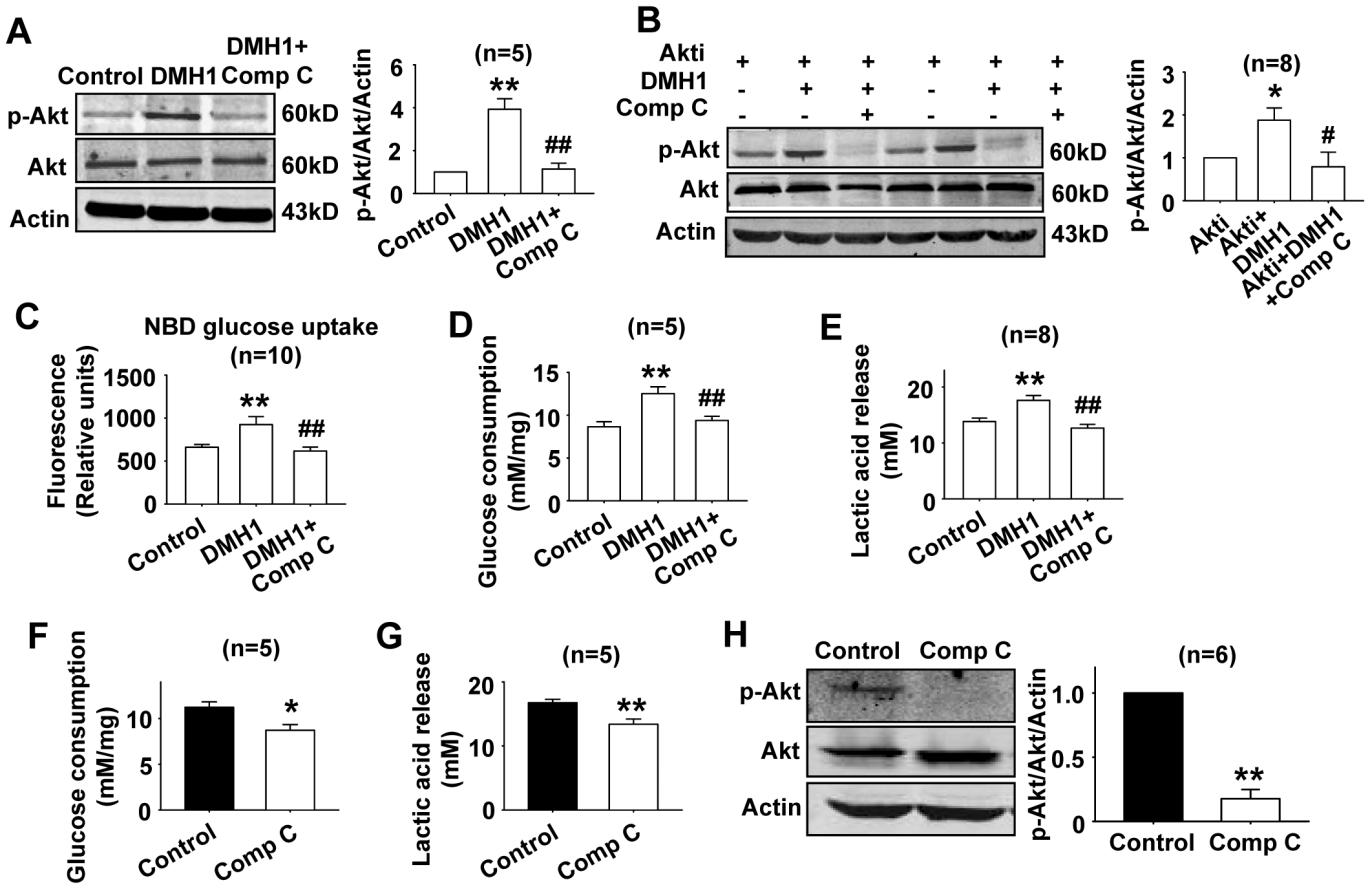
Compound C inhibited DMH1-induced activation of Akt, increases of glucose uptake, glucose consumption, and lactic acid release. (A) Western blot results showed that compound C inhibited DMH1-induced Akt activation in L6 cells. **P <0.01 vs control; ## *P*<0.01 vs. DMH1. (B) Compound C completely inhibited Akt activated by DMH1 in the presence of Akt inhibitor. *P<0.05 vs Akti; #*P*<0.05 *vs* Akti+DMH1. (C–E) Compound C inhibited DMH1-induced increases of glucose uptake, glucose consumption, and lactic acid release. **P<0.01 *vs* control. # *P*<0.05, ## *P*<0.01 vs DMH1. (F–H) Compound C alone inhibited glucose consumption, lactic acid release and p-Akt level in L6 cells. *P<0.05, **P<0.01 *vs* control. The concentrations of DMH1, compound C and Akti were 10 µM, 10 µM, and 0.5 µM respectively. Akti, Akt inhibitor, (1,3-Dihydro-1-(1-((4-(6-phenyl-1H -imidazo[4,5-g]quinoxalin-7-yl)phenyl)methyl)-4-piperidinyl)-2H-benzimidazol-2-one trifluoroacetate salt hydrate).

Compound C was reported to block Akt pathway in cancer cells [3]. Here, we found compound C inhibited DMH1-induced Akt activation in L6 cells (Figure.6A). Next, we treated L6 cells with DMH1 and DMH1 plus compound C in the presence of Akt inhibitor. Results showed that DMH1 still activated Akt in L6 cells which were pretreated with Akt inhibitor, but when the cells were co-treated with compound C, the activated Akt was almost completely inhibited (Figure.6B).

Then we measured the effects of compound C on DMH1-induced increase of glucose uptake, consumption and lactic acid release. As shown in Figure.6C–6E, compound C inhibited DMH1-induced increase of glucose uptake, consumption and lactic acid release. Compound C alone also inhibited glucose consumption, lactic acid release and p-Akt level in L6 cells (Figure.6F–6H).

### DMH1 induces Akt activation through inhibiting PP2A activity in L6 cells

**Figure 7:**
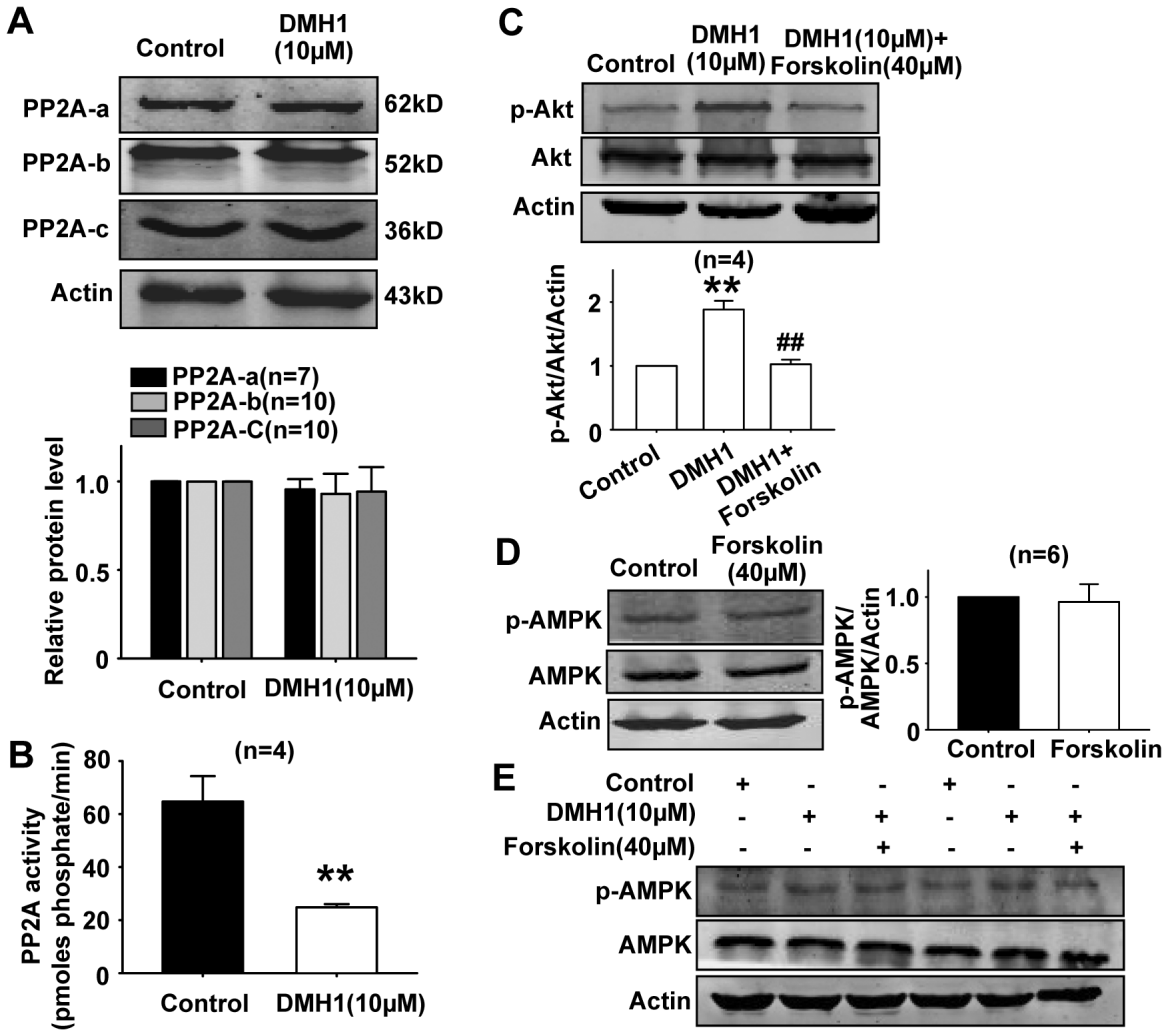
DMH1 induces Akt activation through inhibiting PP2A in L6 cells. (**A**) DMH1 treatment showed no effect on the protein expressions of PP2A-a, PP2A-b, and PP2A-c subunits. (**B**) DMH1 treatment inhibited PP2A activity in L6 cells. **P<0.01 vs control. (**C**) PP2A activator forskolin (40 µM) inhibited DMH1-induced Akt activation in L6 cells. **P<0.01 *vs* control. ## *P*<0.01 vs DMH1.(**D–E**) Forskolin (40 µM) showed no effects on p-AMPK level in L6 cells.

Protein phosphatase 2A (PP2A) is a ubiquitously expressed cytoplasmic serine/threonine phosphatase that plays an important role in regulation of a diverse set of cellular proteins, including metabolic enzymes [17], [18]. PP2A inhibits Akt activation [19]. Since DMH1 activated Akt in L6 cells, we further examined the effects of DMH1 on the expression and activity of PP2A, the upstream signal of Akt, in L6 cells. DMH1 treatment showed no effect on the protein expressions of PP2A-a, PP2A-b, and PP2A-c subunits (Fig.7A), but significantly inhibited PP2A activity in L6 cells (Fig.7B).

In order to identify whether the inhibition of PP2A activity by DMH1 contributes to Akt activation in L6 cells, we used PP2A activator forskolin [20], [21] to reverse DMH1-induced PP2A activity inhibition. As shown in Figure.7C, forskolin significantly inhibited DMH1-induced Akt activation in L6 cells, indicating that DMH1-induced Akt activation was due to PP2A activity inhibition. We further examined the effects of forskolin on AMPK protein expression. Forskolin (40 µM) alone showed no effects on p-AMPK level in L6 cells, even at the presence of DMH1 (Figure.7D–7E).

## Discussion

DMH1 was first developed as a potent and selective inhibitor of BMP signaling [4]. Recent studies reported that DMH1 had multiple effects, for example, DMH1 increased cardiomyocyte progenitors and promotes cardiac differentiation in mouse embryonic stem cells [22], promoted neurogenesis of hiPSCs [23]. Our earlier studies found that DMH1 inhibited pathological cardiac hypertrophy and its related Kv4.3 K^+^ channel remodeling [8], [24]. Here, we found that DMH1 increased glucose metabolism through activating Akt and DMH1 activated Akt through inhibiting PP2A activity in L6 rat muscle cells. The present study provides a novel leading chemical structure targeting Akt with potential use for regulating glucose metabolism.

**Figure 8:**
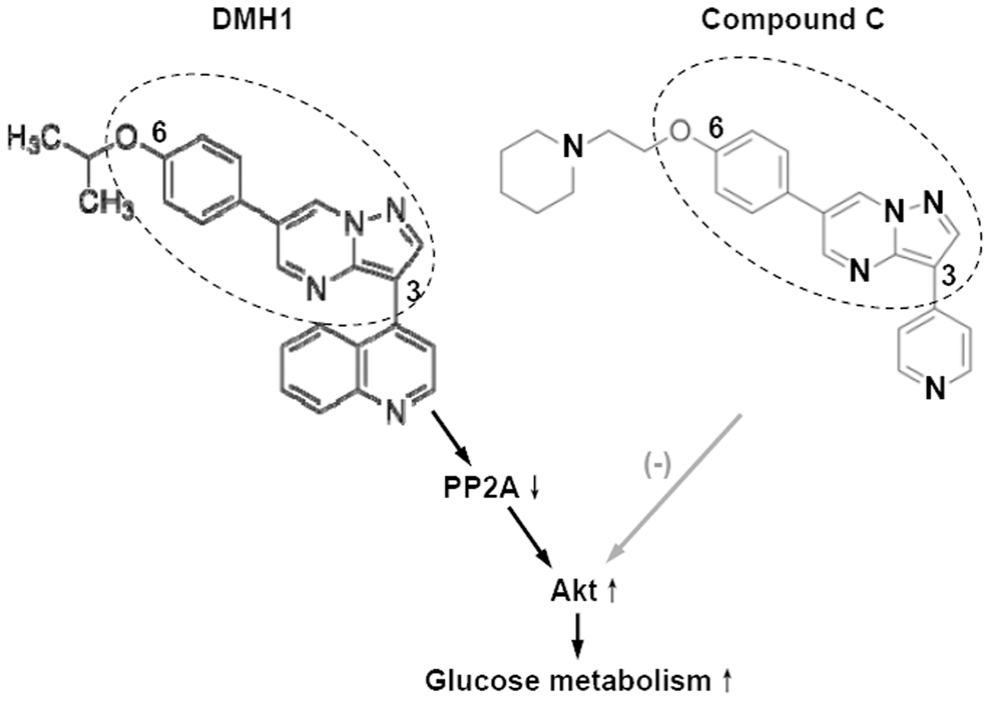
Schematic diagram showing the chemical structure and pharmacological effects of DMH1 and compound C on Akt and glucose metabolism. DMH1 activated Akt and increased glucose metabolism; but compound C inhibited DMH1 –induced Akt activation and glucose metabolism increase.

DMH1 is a compound C analogue. Vucicevic *et al*. had shown that compound C inhibited Akt [3]. By comparing the chemical structure and pharmacological effects of DMH1 and compound C, we summarized the comparison of DMH1 and compound C on Akt and glucose metabolism in Figure.8: DMH1 activated Akt through inhibiting PP2A activity and increased glucose metabolism; but compound C inhibited DMH1 –induced Akt activation and glucose metabolism increase. Based on the similar structure of DMH1 and compound C and their opposite effects on Akt, we postulate the parent structure of DMH1 and compound C is a valuable inspiration for chemists to develop drugs for glucose metabolism regulation and diabetes treatment.

Hyperactivation of PP2A in liver, muscle, retina and the pancreatic islet occurred under the duress of glucolipotoxicity and diabetes [25]. Inhibition of high glucose-induced PP2A hyperactivation prevented NF-κB activity and cell death in bovine aortic endothelial cells, suggesting that PP2A plays critical regulatory roles in hyperglycemia induced cell demise [26]. In primary rat hepatocytes, palmitate incubation resulted in a significant inhibition of insulin-mediated Akt activation and a significant increase in PP2A expression and activity. Pharmacological inhibition of PP2A restored insulin-induced Akt activation in palmitate-treated cells [27]. Based on the pathological role of PP2A hyperactivation in diabetes, we thought that the present work put forward a novel PP2A inhibitor with potential use for mitigating diabetic duress.

Both Akt and AMPK activation lead to increase of glucose metabolism, but it is unnecessary to activate both Akt and AMPK at the same time. For example, ciliary neurotrophic factor increases muscle glucose uptake by a mechanism involving the Akt pathway that does not require AMPK [28]. We found that DMH1 increased glucose metabolism through activating Akt in L6 rat skeletal muscle cells, and Akt inhibitor and compound C inhibited DMH1-induced increase of glucose metabolism. Although compound C is commonly used as AMPK inhibitor, here, we thought that compound C inhibited DMH1-induced increase of glucose metabolism through inhibiting Akt.
